# Amphiregulin induces CCN2 and fibronectin expression by TGF-β through EGFR-dependent pathway in lung epithelial cells

**DOI:** 10.1186/s12931-022-02285-2

**Published:** 2022-12-28

**Authors:** Wun-Hao Cheng, Shih-Ya Kao, Chia-Ling Chen, Fara Silvia Yuliani, Lee-Yuan Lin, Chien-Huang Lin, Bing-Chang Chen

**Affiliations:** 1 School of Respiratory Therapy, College of Medicine, Taipei Medical University, 250 Wu-Hsing Street, Taipei, 11031 Taiwan; 2 Respiratory Therapy, Division of Pulmonary Medicine, Department of Internal Medicine, Wan Fang Hospital, Taipei Medical University, Taipei, Taiwan; 3 International Graduate Program in Medicine, College of Medicine, Taipei Medical University, Taipei, Taiwan; 4 Department of Pharmacology and Therapy, Faculty of Medicine, Public Health, and Nursing, Universitas Gadjah Mada, Yogyakarta, Indonesia; 5 School of Medicine, Collage of Medicine, Taipei Medical University, Taipei, Taiwan; 6 Gradual Institute of Medical Sciences, College of Medicine, Taipei Medical University, 250 Wu-Hsing Street, Taipei, 11031 Taiwan; 7 Division of Thoracic Medicine, Department of Internal Medicine, School of Medicine, College of Medicine, Taipei Medical University, Taipei, Taiwan

**Keywords:** Severe asthma, Amphiregulin, TGF-β, CCN2, EMT, Airway fibrosis, EGF, EGFR

## Abstract

**Background:**

Airway fibrosis is one of the pathological characteristics of severe asthma. Transforming growth factor (TGF)-β has been known to promote epithelial-mesenchymal transition formation and to play a role in the progression of tissue fibrosis. Cellular communication network factor 2 (CCN2) and fibronectin (FN) are well-known markers of EMT and fibrosis. However, whether AREG is involved in TGF-β-induced CCN2 and FN expression in human lung epithelial cells is unknown.

**Methods:**

AREG and FN were analyzed by immunofluorescence staining on ovalbumin-challenged mice. CCN2 and FN expression were evaluated in human lung epithelial (A459) cells following TGF or AREG treatment for the indicated times. Secreted AREG from A549 cells was detected by ELISA. Cell migration was observed by a wound healing assay. Chromatin immunoprecipitation was used to detect the c-Jun binding to the CCN2 promoter.

**Results:**

AREG and FN expression colocalized in lung tissues from mice with ovalbumin-induced asthma by immunofluorescence staining. Moreover, TGF-β caused the release of AREG from A549 cells into the medium. Smad3 siRNA down-regulated AREG expression. AREG also stimulated CCN2 and FN expression, JNK and c-Jun phosphorylation, and cell migration in A549 cells. AREG small interfering (si) RNA inhibited TGF-β-induced expression of CCN2, FN, and cell migration. Furthermore, AREG-induced CCN2 and FN expression were inhibited by EGFR siRNA, a JNK inhibitor (SP600125), and an activator protein-1 (AP-1) inhibitor (curcumin). EGFR siRNA attenuated AREG-induced JNK and c-Jun phosphorylation. Moreover, SP600125 downregulated AREG-induced c-Jun phosphorylation.

**Conclusion:**

These results suggested that AREG mediates the TGF-β-induced EMT in human lung epithelial cells through EGFR/JNK/AP-1 activation. Understanding the role of AREG in the EMT could foster the development of therapeutic strategies for airway remodeling in severe asthma.

## Introduction

Asthma affected about 262 million people and caused 461,000 deaths around the world in 2019 [1]. Airway remodeling, inflammation, and lung fibrosis are common pathological features of severe asthma [2, 3]. Lung fibrosis results in fibroblast proliferation, extracellular matrix (ECM) deposition (e.g., fibronectin (FN) and collagen), and alveolar epithelial-mesenchymal transition (EMT) [4]. Moreover, transforming growth factor (TGF)-β is a well-known cytokine in the process of airway inflammation and remodeling, as well as EMT [4, 5]. However, there are few therapeutic strategies for the alveolar EMT in severe asthma.

Amphiregulin (AREG) is a membrane protein that is a member of the epidermal growth factor (EGF) family. In addition, AREG can be proteolyzed by a disintegrin and metalloprotease 17 (ADAM17), which results in a soluble form of the ligand [6–8]. Soluble AREG acts as a ligand for the EGF receptor (EGFR) and is responsible for cell survival, growth, and migration [7, 9]. Previous studies showed that AREG participates in inflammation and injury in different tissues such as the lungs, liver, and intestines [10–12]. AREG was found to lead to the proliferation of airway epithelial cells and airway smooth muscle cells through EGFR activation, thereby affecting airway remodeling in severe asthma [13, 14].

EMT is a process of epithelial cell transition into mesenchymal cells in tissue injury that leads to organ fibrosis [15]. Fibroblast-like mesenchymal cells can express FN, collagens, and α-smooth muscle actin (α-SMA) as EMT markers [15, 16]. Previous studies have shown that TGF-β can induce EMT by activating either the classical SMAD 2/3 or a non-classical signaling pathway [17, 18]. Increasing evidence has revealed that EMT of alveolar epithelial cells can be observed in lung fibrosis [4, 15]. However, the role of AREG in the EMT of alveolar tissues is still unknown.

Cellular communication network factor 2 (CCN2), a fibrotic marker, is a well-known mediator of tissue remodeling and fibrosis [19]. Numerous studies have reported that increased CCN2 can induce ECM deposition, myofibroblast differentiation, and EMT [20–22]. CCN2 expression has been demonstrated to be increased by several stimulators such as TGF-β, hypoxia, and vascular endothelial growth factor in lung fibroblasts [19, 21, 23]. Thus, CCN2 plays a crucial role during tissue fibrosis or EMT. Nonetheless, the mechanism of AREG-induced CCN2 expression has yet to be identified.

Activator protein (AP)-1, a c-Jun/c-Fos complex, is a c-Jun N-terminal kinase (JNK) downstream transcription factor. The JNK/AP-1 pathway is involved in regulating cell proliferation, differentiation, survival, and apoptosis [24, 25]. Our previous study showed that JNK and AP-1 participate in lung fibrosis [21]. Also, AREG can activate the phosphorylation of mitogen-activated protein kinase (MAPK) in the abnormal proliferation of vascular smooth cells [26]. However, whether JNK/AP-1 mediates the AREG-induced EMT needs to be identified.

In this study, we found that AREG and FN were co-expressed in the lung tissues of asthmatic mice. Moreover, AERG induced CCN2 expression in A549 cells via the EGFR/JNK/AP-1 pathway.

## Materials and methods

### Materials

Dulbecco's modified Eagle medium (DMEM)/F-12 (12,500,062), Opti medium (31,985,070), and antibiotic–antimycotic (100 ×) (15,240,062) were purchased from GIBCO (Waltham, MA, USA). Control small interfering (si)RNA (scrambled), AREG siRNA, EGFR siRNA, Smad3 siRNA, fetal bovine serum (FBS) (F2442), a human AREG enzyme-linked immunosorbent assay (ELISA) kit (RAB0019), and an α-tubulin antibody were obtained from Sigma-Aldrich (St. Louis, MO, USA). Lipofectamine 3000 reagent, Lipofectamine Plus reagent (L3000015), and an AREG antibody (PA5-109404) were purchased from Invitrogen Life Technologies (Carlsbad, CA, USA). Recombinant human TGF-β (100-21) and recombinant human AREG (100-55B) were obtained from PeproTech (Cranbury, NJ, USA). An FN (ab2413) antibody and mounting medium with DAPI (ab104139) were purchased from Abcam (Cambridge, UK), while c-Jun phosphorylated (phospho) at Ser63 (#9261), JNK (#9252), phospho-JNK (Thr183/Tyr185) (#9251), and CCN2 (#86641) were purchased from Cell Signaling Technology (Danvers, MA, USA). A phospho-EGFR (Tyr1086) antibody (36-9700) was purchased from ThermoFisher Scientific (Waltham, MA, USA). EGFR (sc-373746) and c-Jun (sc-74543) were purchased from Santa Cruz Biotechnology (Dallas, TX, USA). Goat anti-rabbit immunoglobulin G (IgG) (H + L)-horseradish peroxidase (HRP) (C04003), and goat anti-mouse IgG (H + L)-HRP (C04001) were acquired from Croyez Bioscience (Taipei, Taiwan).

### Cell culture

Human lung epithelial cells (A549) were acquired from American Type Culture Collection (Manassas, VA, USA). Cells were grown in a DMEM/F-12 nutrient mixture, which contained 10% FBS, 100 U/ml penicillin G, and 100 μg/ml streptomycin, in a humidified incubator with 5% CO_2_ and 95% N_2_ at 37 °C. After A549 cells reached 80% confluence, cells were seeded into 6-cm dishes for immunoblotting.

### OVA-induced asthma in mice

The 6–8-week-old C57B/6 female mice were intraperitoneally injected with 200 μl of 50 μg OVA combined with the 4 mg of aluminum hydroxide in phosphate-buffered saline (PBS) on days 0, 7, and 14. After sensitization with OVA, mice were challenged with OVA aerosol (5% in PBS) or PBS aerosol for 30 min, 2 days/week for 9 weeks.

### siRNA transfection

Cells were transfected for 24 h with control siRNA, AREG siRNA, ADAM17, or EGFR siRNA (50 nM) using Lipofectamine^®^ 3000 reagent with Opti medium. Cells were treated with TGF-β or AREG (10 ng/ml) at the indicated time intervals after 24 h of siRNA transfection. Results were analyzed by Western blotting.

### ChIP assay

A549 cells were exposed to AREG (10 ng/ml) for 30 min and fixed with formaldehyde for 10 min. AP-1 binding to the CCN2 promoter region was detected through a ChIP assay, as previously described [21]. A polymerase chain reaction (PCR) of the AP-1 response elements on the CCN2 promoter region was performed using the following primers: 5′- TTC TGG AAA CAT TGA TGG -3′ (sense) and 5′- TAT AGG CTC TTG AAA CTC -3′ (antisense).

### ELISA

The concentrations of AREG in the culture medium were determined by ELISA Sigma-Aldrich (St. Louis, MO, USA) according to the manufacturer's instructions. Following TGF-β stimulation for the indicated time, 100 μl of culture medium was added to a 96-well plate coated with AREG capture antibody. After 4 washes with wash buffer, 100 μl of AREG detection antibody was added to a 96-well plate at room temperature with shaking at 500 rpm for 1 h. Following 4 additional washes, 100 μl of conjugate antibody (HRP) was added for 45 min. After the final 4 washes, 100 μl of substrate buffer was added for 20 min in the dark. The reaction was terminated with stop solution. The relative absorbance was then measured using an ELISA reader to represent the concentration.

### Western blotting

The Western blot analysis was as previously described [27]. Cells were cultured in 6-cm dishes. After cell lysis and protein extraction from A549 cells, proteins were transferred to a polyvinylidene difluoride (PVDF) membrane from sodium dodecylsulfate polyacrylamide gel electrophoresis (SDS-PAGE). The PVDF membrane was blocked by 5% bovine serum albumin (BSA) and then incubated with a specific primary antibody and an HRP-conjugated secondary antibody. Enhanced chemiluminescence was used to detect the immunoreactivity. The intensity of the signal was quantified using Image-Pro software (Eastman Kodak, Rochester, NY, USA).

### Immunofluorescence (IF) staining

Serial 4-μm paraffin sections from OVA- or PBS-treated mice were used to detect AREG and FN by IF staining, as described previously [27]. In brief, slides were incubated with PBS containing 0.25% Triton X-100 for permeabilization. Slides were blocked with 5% BSA and incubated with specific antibodies such as AREG (PA5-109,404; Invitrogen, 1:1000) or FN (ab2413; Abcam, 1:1000) in 1% BSA overnight, and were in turn incubated with a fluorescein isothiocyanate (FITC)-conjugated secondary antibody for 1 h in the dark at room temperature. Slides were incubated with 4′,6-diamidino-2-phenylindole (DAPI) staining (ab104139; Abcam) for counter staining. Fluorescent images were captured under a confocal fluorescence microscope (Leica TCS SP5, Wetzlar, Germany).

### Cell migration assay

Cell migration was observed by the wound-healing assay. Wound-healing experiments were analyzed as previous described [28]. Cells were seeded into Culture-Insert 2 Well 24 (Ibidi, Gräfelfing, Germany). After 24 h, the Culture-Insert 2 Well was removed, and the cells were treated with TGF-β or AREG (10 ng/ml) for another 24 h at 37 °C with 5% CO_2_. The images of the wound area were observed by TS100 microscopy (Nikon, Tokyo, Japan), and captured by Lead View 2000AIO (Nikon, Tokyo, Japan). The area of square wound was quantified by image J software.

### Study approval

All animal protocols were approved by the Animal Ethics Committee of Taipei Medical University (approval no. LAC-2016-0361).

### Statistical analysis

Results were analyzed by one-way analysis of variance (ANOVA) with Dunnett's test as a post-test to compare at least three groups. An unpaired-sample *t*-test was performed to compare between two groups. Cell migration assay was analyzed using two-way ANOVA with Bonferroni’s multiple comparisons test. Data are presented as the mean ± standard error of the mean (SEM), and a *p* value of < 0.05 was considered statistically significant.

## Results

### Overexpression of AREG in OVA-induced lung fibrosis in mice

**Fig. 1:**
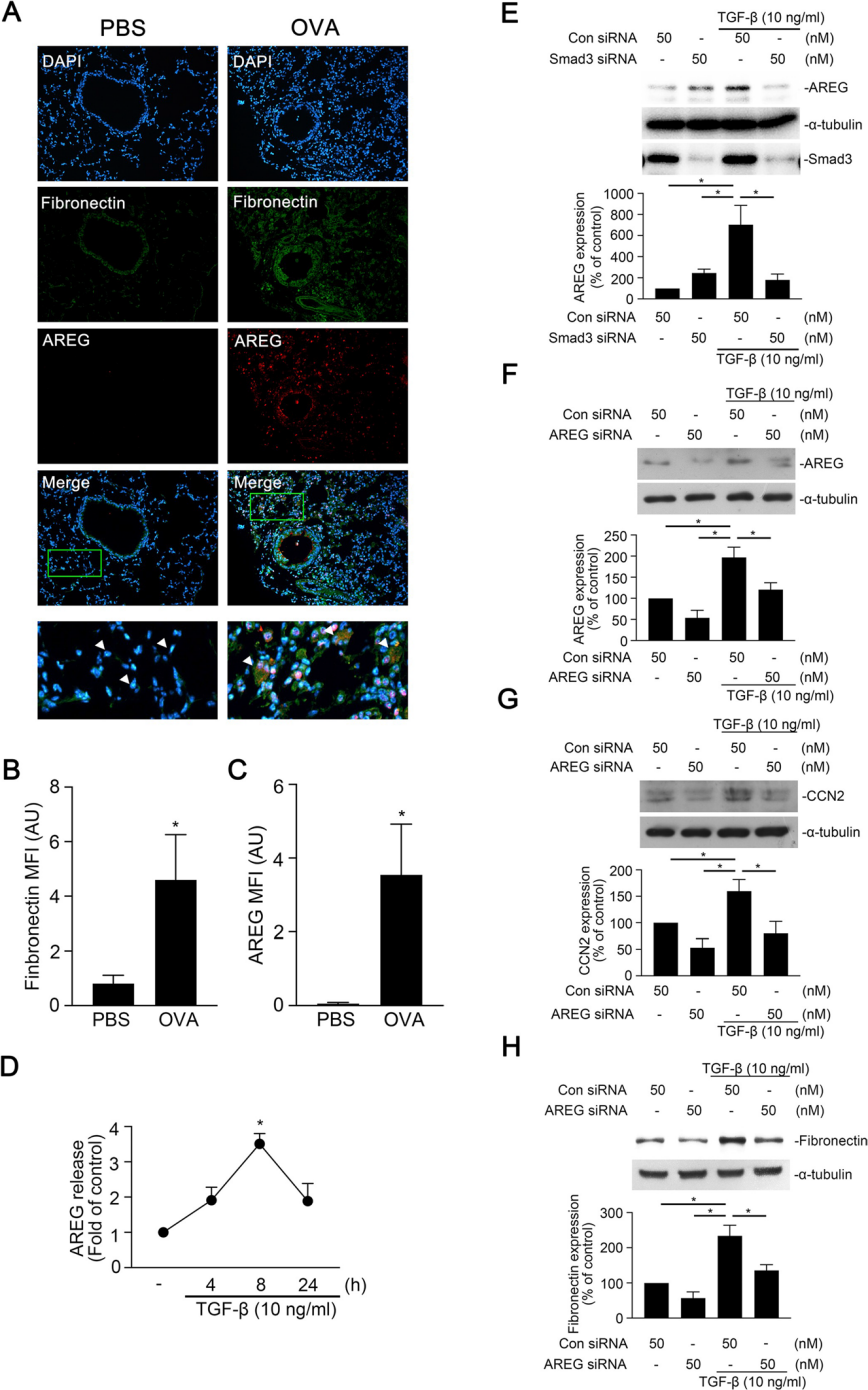
Amphiregulin (AREG) is involved in transforming growth factor (TGF)-β-induced cellular communication network factor 2 (CCN2) and fibronectin expression in A549 human lung epithelial cells. **A** Lung tissue sections from ovalbumin (OVA)- or phosphate-buffered saline (PBS)-treated C57B/L6 mice were observed by immunofluorescence staining. Representative images of AREG (red) and fibronectin (green) from PBS- (*n* = 6) and OVA-treated (*n* = 5) mice. Nuclei were detected by 4′,6-diamidino-2-phenylindole (DAPI) staining (blue) (original magnification, 20 ×). Quantification of **(B)** fibronectin and **(C)** AREG from Fig. 1A. Data are presented as the mean ± SEM (PBS, *n* = 6 and OVA, *n* = 5). *p < 0.05, relative to PBS-treated mice. **D** A549 cells were stimulated with transforming growth factor (TGF)-β (10 ng/ml) for 0 ~ 24 h. Amounts of AREG in supernatants were investigated by an AREG-ELISA. Results are presented as the mean ± SEM, *p < 0.05, *n* = 3, compared to the control group. Furthermore, cells were transfected with 50 nM of control siRNA (con siRNA), Smad3 siRNA, or AREG siRNA for 24 h, and then stimulated with TGF-β (10 ng/ml) for the indicated time. Levels of **(E **and**  F)** AREG, α-tubulin, **(G)** CCN2, α-tubulin, **(H)** fibronectin, and α-tubulin in cell lysates were detected by immunoblots. The results are expressed as the mean ± SEM, *p < 0.05, *n* = 3, relative to the TGF-β-stimulated with the control siRNA group

To identify the role of AREG in lung fibrosis in mice with OVA-induced asthma, we observed thickened interstitial alveoli and airway remodeling in sections of lung tissues from OVA-challenged mice. Moreover, FN and AREG expressions were higher and colocalized in OVA-challenged mice according to dual-label IF staining (Fig. 1A–C). TGF-β also induced the release of AREG from A459 cells with a peak at 8 h (Fig. 1D). Smad3 siRNA (50 nM) attenuated TGF-β-induced AREG expression (Fig. 1E). Moreover, we used AREG siRNA (50 nM) to investigate the role of AREG in TGF-β-induced fibrosis. After transfection of A549 cells with AREG siRNA, we found that AREG siRNA had a functional effect of reducing AREG expression (Fig. 1F). Furthermore, AREG siRNA inhibited TGF-β-induced CCN2 and FN expression (Fig. 1G, H).

### AREG induced fibrotic protein expression in human alveolar epithelial cells

**Fig. 2:**
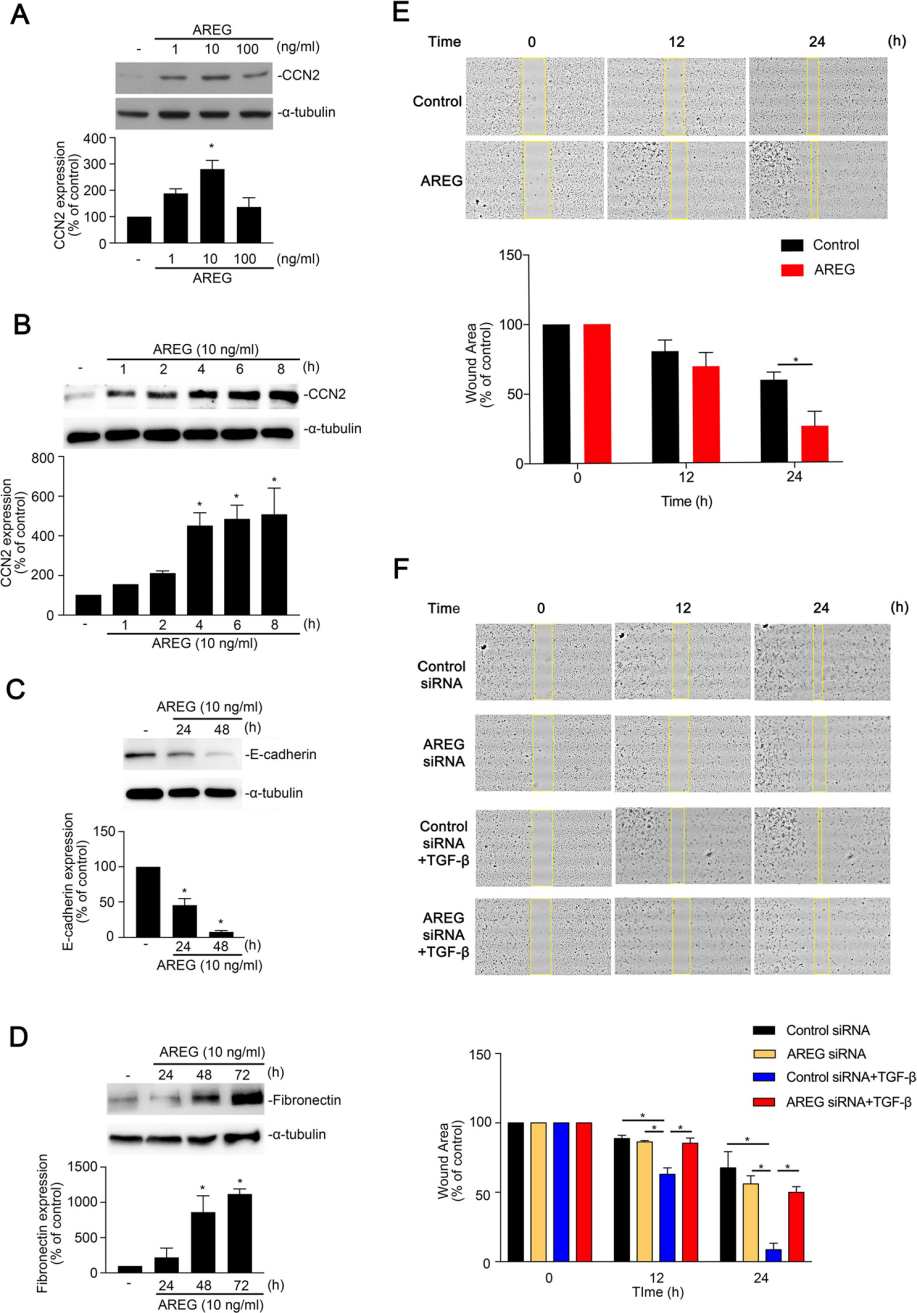
Amphiregulin (AREG)-induced cellular communication network factor 2 (CCN2) and fibronectin expressions in A549 human lung epithelial cells. **A** A549 cells were stimulated with 0 ~ 100 ng/ml AREG for 6 h, and the expression of communication network factor 2 (CCN2) reached its highest level with 10 ng/ml AREG. Cell lysates were prepared and immunodetected with specific antibodies for CCN2 and α-tubulin. Results are expressed as the mean ± SEM, *p < 0.05, *n* = 3, compared to the group without AREG treatment. **B** Cells were incubated with AREG (10 ng/ml) for 0 ~ 8 h, and then the CCN2 and α-tubulin proteins were determined by immunoblots. Data are presented as the mean ± SEM of three experiments. *p < 0.05, compared to the control. Cells were treated with AREG (10 ng/ml) for the indicated time interval. E-cadherin and α-tubulin proteins, **(C)** Fibronectin and α-tubulin proteins **(D)** were determined by immunoblots. Data are presented as the mean ± SEM of three experiments. *p < 0.05, compared to the control. **E** Cells were treated with AREG (10 ng/ml) for 0 ~ 24 h, and wound area (yellow square area) was quantified by image J. Data are presented as the mean ± SEM, *n* = 3. *p < 0.05, compared to the control. **F** Cells were transfected with AREG siRNA (50 nM) for 24 h, and then incubated with TGF-β (10 ng/ml) for 0 ~ 24 h. The wound area (yellow square area) was quantified by image J. Data are presented as the mean ± SEM, *n* = 3. *p < 0.05, relative to the TGF-β-stimulated with the control siRNA group

Since AREG is highly expressed in lung sections from OVA-treated mice, we hypothesized that AREG can induce expressions of fibrotic proteins such as CCN2 and FN in human alveolar epithelial cells. As illustrated in Fig. 2A and B, AREG upregulated CCN2 expression in the time- and dose-dependent manners in A549 cells. Moreover, treatment with AREG (10 ng/ml) decreased the level of E-cadherin and induced FN protein expression in a time-dependent manner (Fig. 2C, D). We also performed a wound-healing assay to assess the role of AREG in the TGF-β-induced EMT process. Figure 2E showed that AREG (10 ng/ml) promoted cell migration compared to the control group at 24 h. AREG siRNA attenuated TGF-β-induced A549 cell migration (Fig. 2F).

### Involvement of the EGFR in AREG-induced activation of JNK/c-Jun and expressions of fibrotic proteins in human alveolar epithelial cells

**Fig. 3:**
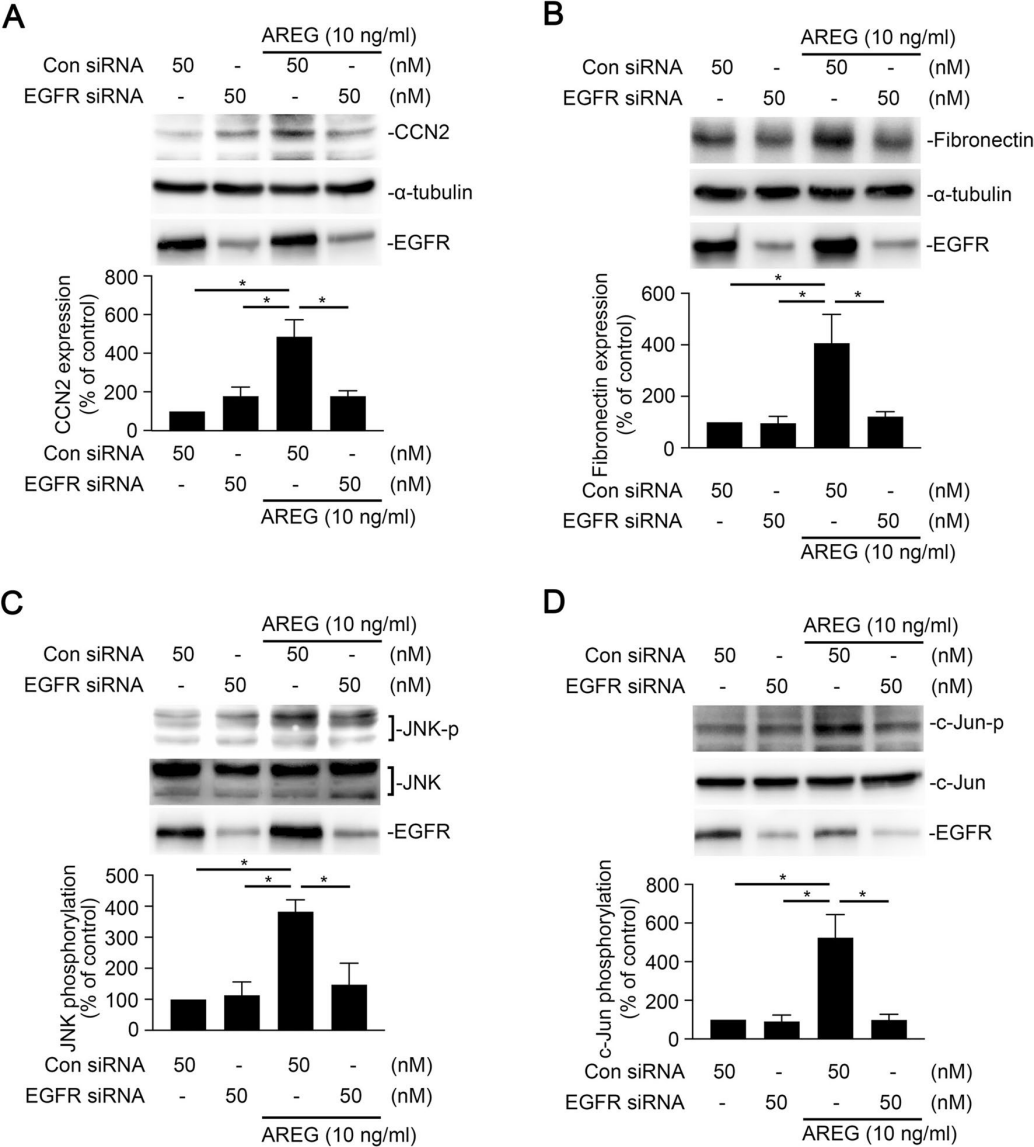
Epidermal growth factor receptor (EGFR) mediates amphiregulin (AREG)-induced cellular communication network factor 2 (CCN2) and fibronectin expressions in A549 human epithelial cells. Cells were transfected with 50 nM of control siRNA (Con siRNA) and EGFR siRNA. After 24 h, cells were stimulated with AREG (10 ng/ml) for the indicated time. Cell lysates were detected using Western blotting and immunodetected with specific antibodies for **(A)** levels of CCN2, α-tubulin, **(B)** fibronectin, α-tubulin, **(C)** phospho-c-Jun N-terminal kinase (JNK) (Thr183/Tyr185), JNK, **(D)** phospho-c-Jun (serine 63), and c-Jun. Results are expressed as the mean ± SEM, *p < 0.05, *n* = 3, relative to the AREG-stimulated with the control siRNA group

To confirm whether EGFR is associated with the AREG-induced EMT through the JNK/c-Jun signaling pathway in human alveolar epithelial cells, we analyzed AREG-induced CCN2 and FN expressions after transfection of EGFR siRNA (50 nM) into A549 cells. As shown in Fig. 3A and B, transfection of EGFR siRNA reduced AREG-induced CCN2 and FN expressions. Similarly, EGFR siRNA decreased AREG-induced phosphorylation of JNK/c-Jun (Fig. 3C, D).

### Involvement of JNK in APRG-induced CCN2 expression in human alveolar epithelial cells

**Fig. 4:**
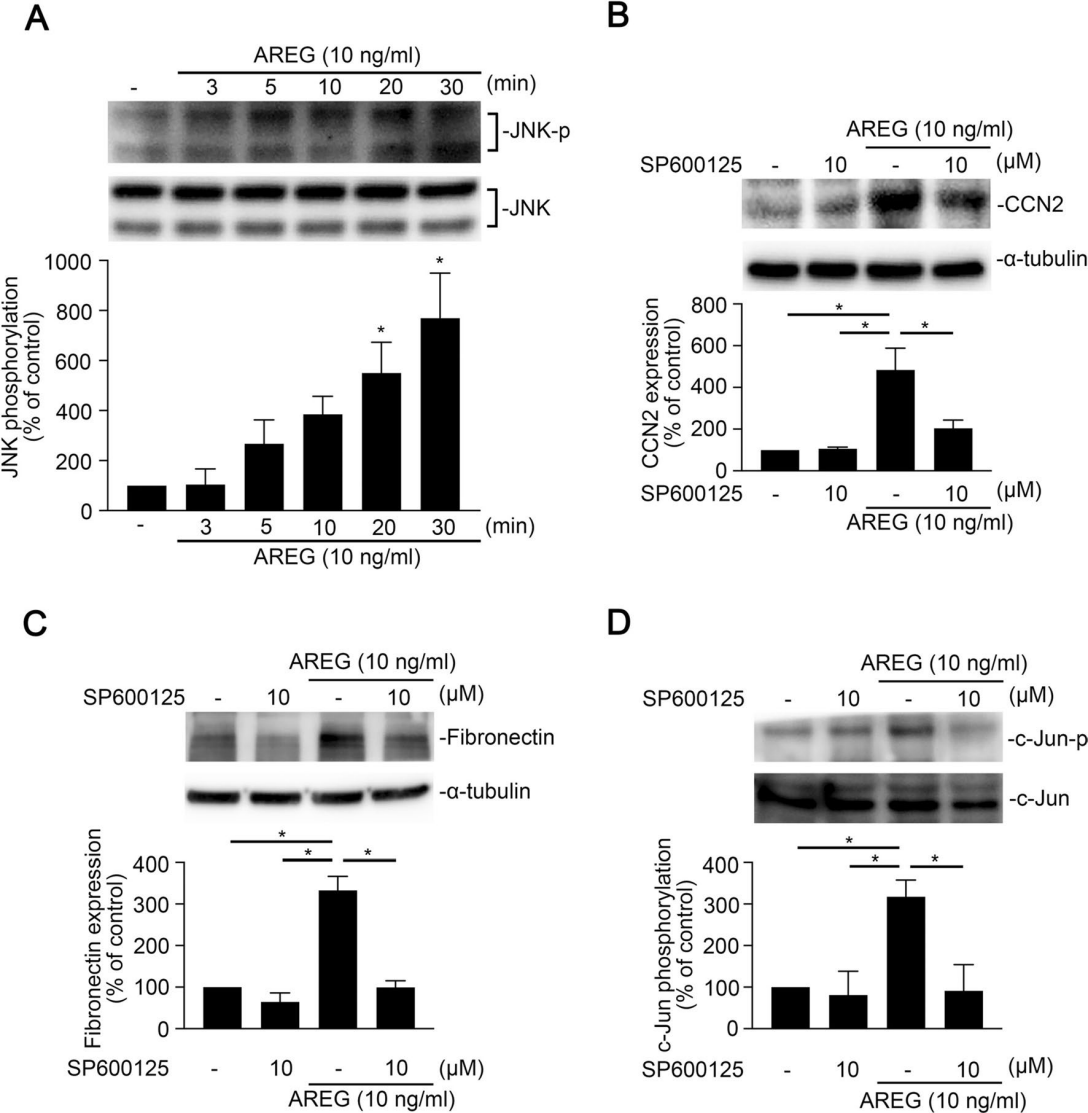
c-Jun N-terminal kinase (JNK) regulates amphiregulin (AREG)-induced cellular communication network factor 2 (CCN2) and fibronectin expressions in A549 human epithelial cells. **A** A549 cells were exposed to AREG (10 ng/ml) for 0 ~ 30 min. Levels of phospho-JNK (Thr183/Tyr185) and JNK in cell lysates were assessed by immunoblotting. Data are shown as the mean ± SEM, *n* = 3. *p < 0.05, relative to unstimulated cells. A549 cells were pretreated with SP600125 (10 μM) for 30 min and then treated with AREG (10 ng/ml) for the indicated time. Levels of **(B)** phospho-c-Jun (serine 63), and c-Jun, *n* = 3, **(C)** fibronectin, α-tubulin, *n* = 4, **(D)** CCN2, α-tubulin, *n* = 3. were detected by immunoblotting. Data are shown as the mean ± SEM, *p < 0.05, relative to AREG-treated cells

To identify the role of JNK in AREG-induced CCN2 expression, we treated A549 cells with 10 ng/ml AREG for various time intervals. AREG significantly provoked JNK Thr183/Tyr185 phosphorylation at 20 and 30 min (Fig. 4A). As illustrated in Fig. 4B, the JNK inhibitor, SP600125 (10 μM), reversed phosphorylation of c-Jun at Ser 63 by AREG in A549 cells. Furthermore, AREG-induced FN and CCN2 expression were inhibited by SP600125 (10 μM) (Fig. 4C, D).

### Mediation of c-Jun in AREG-induced CCN2 expressions in human alveolar epithelial cells

**Fig. 5:**
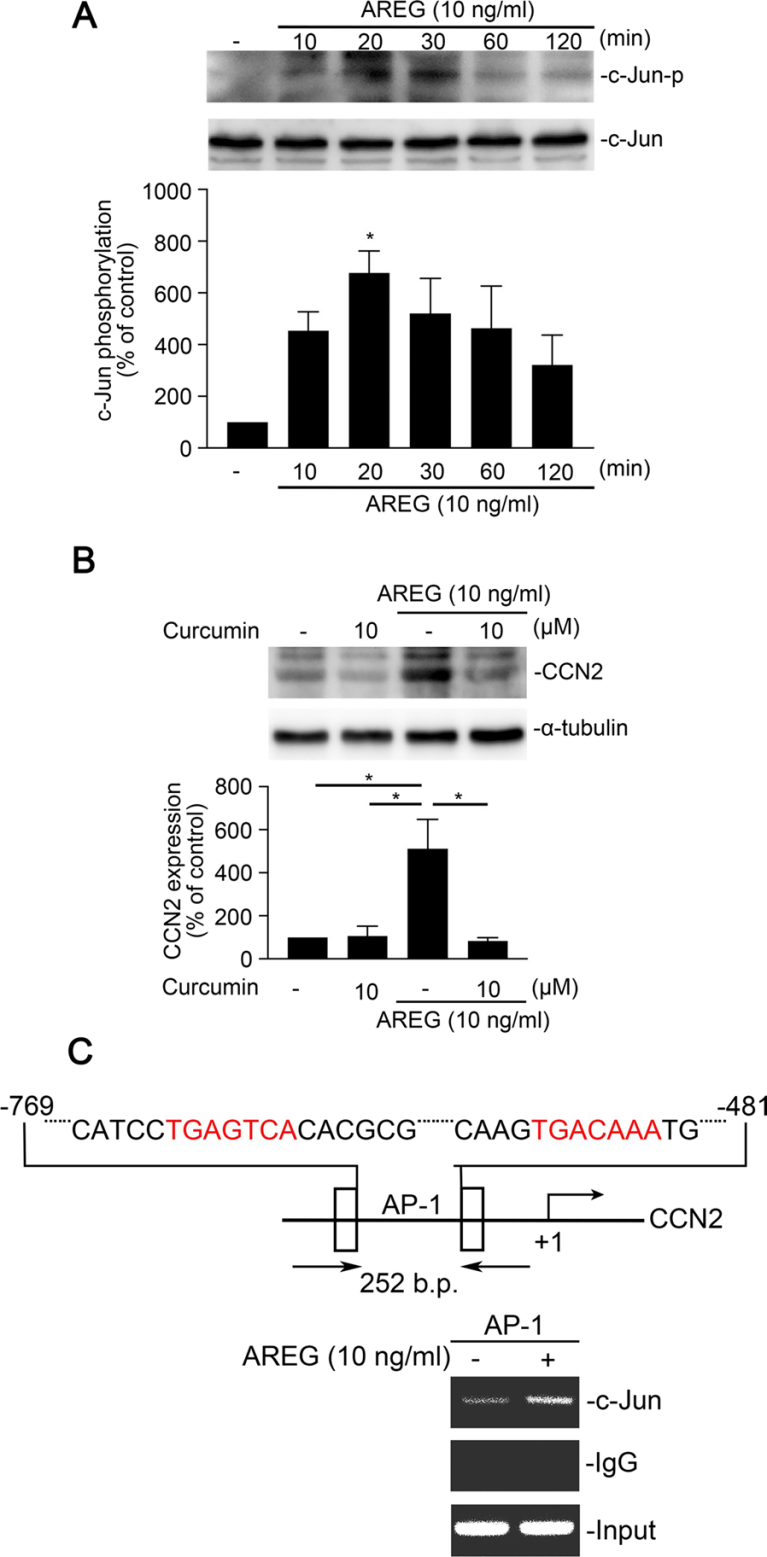
c-Jun is involved in amphiregulin (AREG)-induced cellular communication network factor 2 (CCN2) and fibronectin expressions in A549 human epithelial cells. **A** A549 cells were incubated with AREG (10 ng/ml) for 0 ~ 120 min. Levels of phospho-c-Jun Ser63, and c-Jun were immunodetected with specific antibodies and assessed using Western blotting. The results are expressed as the mean ± SEM, *p < 0.05, *n* = 3, compared to the control group. **B** Cells were pretreated with curcumin for 30 min and then stimulated with AREG (10 ng/ml) for another 6 h. Western blotting was performed to assess the levels of cellular communication network factor 2 (CCN2) and α-tubulin in cell lysates, Data are shown as the mean ± SEM, *n* = 3. *p < 0.05, relative to AREG-stimulated cells. **C** Schematic diagram of activator protein (AP)-1 binding to the CCN2 promoter. A549 cells were exposed to AREG (10 ng/ml) for 30 min. The AP-1-binding site of the CCN2 promoter region was identified through a ChIP assay. Typical traces are presented in all three experiments

C-Jun is a well-known substrate for JNK phosphorylation [29]. A549 cells were stimulated with AREG (10 ng/ml) for 0, 10, 20, 30, 60, and 120 min to examine the role of c-Jun Ser63 in the process of AREG-induced EMT and CCN2 expression, and the phosphorylation of c-Jun was examined using a Western blotting. AREG stimulated c-Jun phosphorylation at Ser63 at 20 min in A549 cells (Fig. 5A). Then, we treated curcumin, an AP-1 inhibitor, to understand whether c-Jun is involved in the AREG-induced CCN2 expression in A549 cells. Figure 5B showed that curcumin decreased AREG-induced CCN2 expression. Furthermore, AREG induced c-Jun binding to the promoter of *CCN2* in A549 cells (Fig. 5C).

## Discussion

Severe asthma results in goblet cell hyperplasia, airway remodeling, and lung inflammation, which in turn induces lung fibrosis [2, 30]. In particular, the alveolar EMT plays an important role in lung fibrosis [4, 15, 31, 32]. Furthermore, AREG can induce fibroblast proliferation and the expression of fibrotic genes (such as collagen 1-α1, α-SMA, and FN) [6]. In this study, we demonstrated that expressions of AREG and FN increased in lung sections from OVA-sensitized mice, which were used as an asthma model. Moreover, colocalization of AREG and FN was observed in alveolar tissues. This indicated that AREG was associated with lung fibrosis and the alveolar EMT in asthmatic mice. AREG siRNA inhibited TGF-β-induced CCN2 and FN expression in A549 cells. Taken together, these results revealed that AREG mediated TGF-β-induced alveolar EMT and contributed to asthma aggravation.

The EMT is a crucial process during fibrogenesis. Numerous studies have revealed that TGF-β1 induces EMT via multiple pathways (e.g. Smad 2/3, PI3K, and MAPK pathways) [33–35]. Moreover, β-catenin induces vimentin, α-SMA, and collagen-I in A549 alveolar epithelial cells during pulmonary fibrosis [36]. In this study, AREG stimulated the induction of CCN2 and FN in A549 alveolar epithelial cells. In addition, AREG promoted A549 cell migration, and AREG siRNA inhibited TGF-β-stimulated cell migration. Therefore, these results suggest that AREG has a vital role in the EMT process.

A previous study has reported that AREG acts as an important mediator in inflammation and the tissue repair process in lung diseases [37]. Furthermore, higher concentrations of AREG in sputum were observed in various lung diseases, such as cystic fibrosis, chronic obstructive pulmonary disease, and asthma [38–40]. In children with persistent asthma, *Areg* expression was up-regulated in nasal epithelial cells compared bronchial epithelium in RNA-seq analysis [41]. The severity of asthma was correlated with the concentrations of AREG in the sputum and blood [39, 42]. Novali et al. reported that AREG expression was localized in bronchial epithelial cells in airway sections of asthma patients, and was correlated with the severity of asthma [39, 43]. Airway epithelial cells produce IL-33, IL-25, and thymic stromal lymphopoietin (TSLP) under allergen stimulation in the type 2 immune reaction of asthma. These cytokines then induce the release of AREG from group 2 innate lymphoid cells for tissue repair [44]. In addition, the level of *Areg* was increased in OVA-specific ST2hi memory Th2 cells after IL-33 stimulation in RNA-seq data [45]. These results indicated that AREG plays a role in type 2 asthma. In our study, we found that TGF-β could induce the expression and release of AREG in human lung epithelial cells. AREG siRNA downregulated CCN2 expression, and AREG could induce EGFR phosphorylation in A459 cells. Thus, these results suggested that AREG participated in TGF-β-induced CCN2 expression in A459 cells.

CCN2 is a central mediator of tissue fibrosis and EMT [19, 46]. A previous study showed that inhibition of CCN2 could attenuate lung fibrosis in bleomycin-treated mice [47]. Moreover, CCN2 increased collagen and α-SMA expression in human lung fibroblasts through the Rac1/MLK3/JNK/AP-1 pathway [21]. Interestingly, severe acute respiratory syndrome coronavirus-2 infection increased mRNA levels of CCN2, TGF-β, ADAM17, and FN in alveolar epithelial cells [48]. A previous study indicated that CCN2 interacts with FN to induce lung fibrosis via the integrin signaling pathway [49]. In addition, we previously found that CCN2 siRNA could decrease TGF-β-induced FN expression in lung epithelial cells [50]. In the present study, TGF-β induced AREG expression, which in turn stimulated EMT markers expression through the EGFR-dependent pathway in human epithelial cells. However, the mechanism by which CCN2 regulates AREG-induced FN up-regulation still requires further investigation. Taken together, CCN2 plays a role in mediating lung fibrosis. It can be used to develop a strategy for the alveolar EMT in severe asthma.

AREG is shed from epithelial cells by ADAM17, which in turn activates EGFR in fibroblasts [6]. In a previous study, profibrotic protein expression (e.g., TGF-β, CCN2) and airway fibrosis were suppressed in OVA-treated ADAM17^f/f^/Cre^+^ mice [51]. Moreover, TGF-β activates ADAM17, which in turn leads to the EMT via angiotensin-converting enzyme-2 ectodomain shedding or the RSK1/C/EBPβ pathway [50, 52]. A Previous study found that AREG has a synergistic effect on TGF-β-induced EMT via Smad 2/3 pathway in fibroblasts [53]. In this study, Smad3 siRNA could down-regulated TGF-β-induced AREG expression in A549 cells. It is reasonable to speculate that TGF-β-induced AREG expression through Smad pathway, then shedding by ADAM17 to induce EMT process in human epithelial cells. However, the more clear mechanism still needs further investigation.

EGFR expression was positively correlated with the severity of asthma [54]. Zhou et al. revealed that inhibiting EGFR reduced TGF-β-induced pulmonary fibrosis [6]. Furthermore, TGF-β induced AREG expression, which in turn stimulated fibroblast proliferation or myofibroblast transformation through EGFR/PI3K/Akt or MAPK/ERK signaling [53]. In our previous study, the EGFR was found to be involved in fibroblast differentiation and thrombin- or TGF-β-induced ECM production [51]. In this study, we found that EGFR siRNA downregulated AREG-induced CCN2 and FN expressions in human epithelial cells. These results indicated that the AREG-induced alveolar EMT was required for EGFR.

**Fig. 6:**
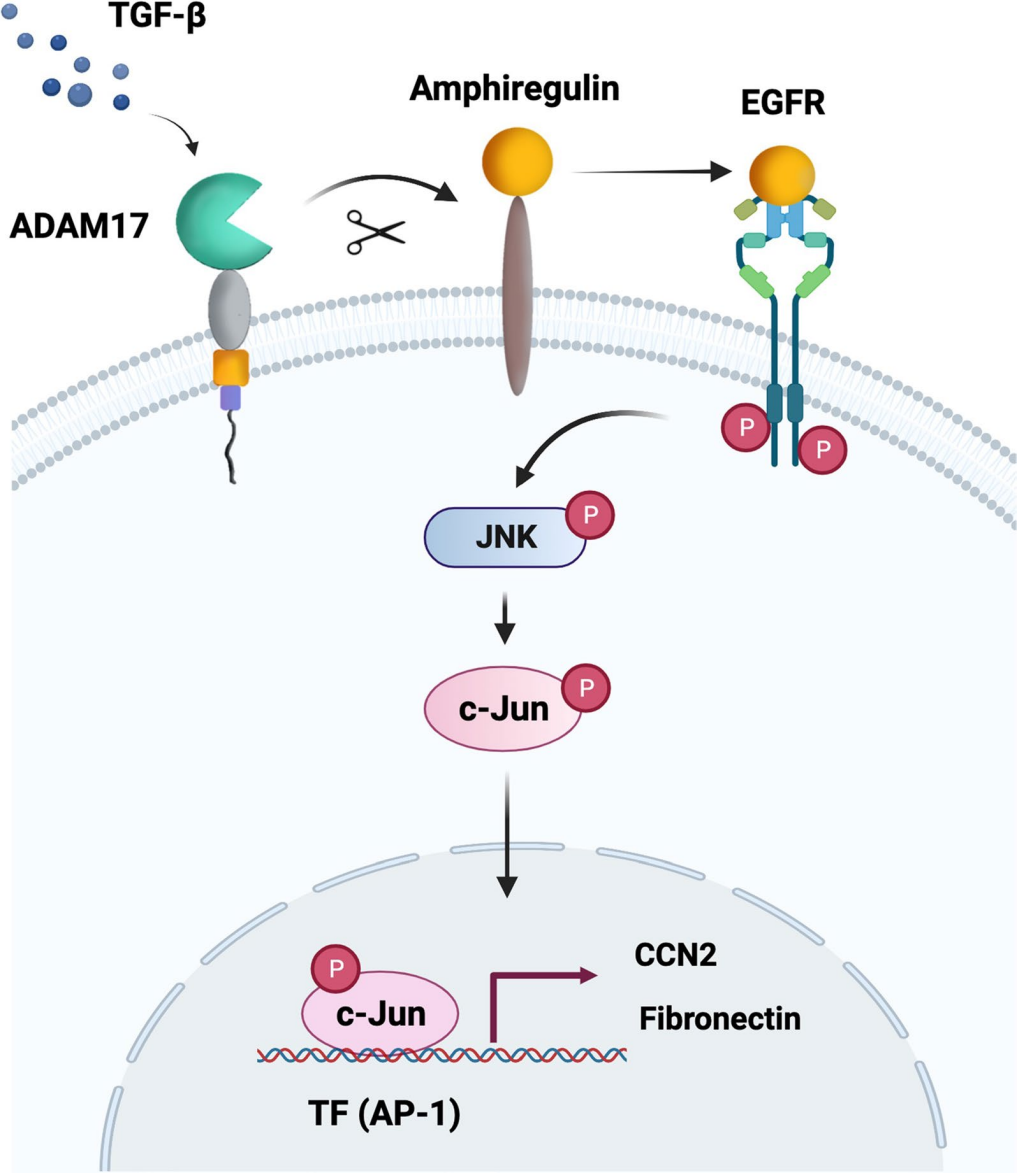
Simplified diagram of the results of amphiregulin (AREG)-induced cellular communication network factor 2 (CCN2) and fibronectin via the epidermal growth factor receptor EGFR/c-Jun N-terminal kinase (JNK)/c-Jun pathway in A549 human lung epithelial cells. Transforming growth factor (TGF)-β induces the release of AREG, which in turn activates EGFR, resulting in the activation of JNK and activator protein (AP)-1. Moreover, AP-1 mediates CCN2 and FN expression through AREG stimulation in A549 human lung epithelial cells, which leads to EMT. Schematic figure created with BioRender.com (https://biorender.com/)

In conclusion, AREG and FN were found to be colocalized in lung sections of asthmatic mice. Moreover, AREG mediates fibrotic protein CCN2 expression through EGFR/JNK/c-Jun signaling in human epithelial cells (Fig. 6). We show for the first time how AREG regulates the expression of CCN2 and alveolar EMT in human lung epithelial cells. This will help develop strategies for the prevention and treatment of lung fibrosis in severe asthma.

## Data Availability

Not applicable.

## Abbreviations

ADAM17: A disintegrin and metalloproteinase 17
AP-1: Activator protein-1
AREG: Amphiregulin
α-SMA: α-Smooth muscle actin
BSA: Bovine serum albumin
CCN2: Cellular communication network factor 2
DMEM: Dulbecco’s modified minimal essential/Eagle medium
ECM: Extracellular matrix
EGFR: Epidermal growth factor receptor
ELISA: Enzyme-linked immunosorbent assay
EMT: Epithelial-mesenchymal transition
FN: Fibronectin
HRP: Horseradish peroxidase
IF: Immunofluorescence
IgG: Immunoglobulin G
IL: Interleukin
JNK: C-Jun N-terminal kinase
MAPK: Mitogen-activated protein kinase
OVA: Ovalbumin
NEAAs: Nonessential amino acids
NHLF: Normal human lung fibroblast
PBS: Phosphate-buffered saline
PCR: Polymerase chain reaction
SDS-PAGE: Sodium dodecylsulfate polyacrylamide gel electrophoresis
siRNA: Small interfering RNA
TGF: Transforming growth factor
TSLP: Thymic stromal lymphopoietin
